# Mechanical and Vibration Performance of Novel Lightweight Sandwich Structures with EPS Beads Filled Syntactic Foam Cores

**DOI:** 10.1021/acsomega.5c09456

**Published:** 2026-02-05

**Authors:** Mehmet Fatih Şansveren, Mustafa Yaman

**Affiliations:** Department of Mechanical Engineering, 37503 Atatürk University, Erzurum 25240, Turkiye

## Abstract

This study introduces a new class of lightweight sandwich composites featuring syntactic foam cores filled with expanded polystyrene (EPS) beads and reinforced by single-layer glass fiber-reinforced polymer (GFRP) face sheets. The hybrid core structure was formulated by embedding hollow glass microballoons (HGMs) and EPS beads of varying densities (10, 18, and 30 kg/m^3^) into an epoxy matrix, enabling precise control over core morphology and mechanical behavior. The structural performance was comprehensively evaluated through uniaxial compression, three-point bending, and free vibration tests. To complement the experimental investigations, a finite element model based on third-order shear deformation theory was developed to simulate the vibrational response. The model exhibited strong agreement with experimental data, confirming its predictive accuracy. Results reveal that increasing EPS bead density significantly enhances the core and overall composite density, leading to improved compressive and flexural strengths, elevated natural frequencies, and reduced damping capacity. Notably, the sandwich architecture dramatically boosted the flexural load-bearing capacity of the syntactic cores—achieving up to a 5-fold improvement over the standalone core materials. These findings underscore the effectiveness of EPS bead-filled syntactic foams in tailoring the mechanical and dynamic properties of sandwich composites. The proposed design strategy offers a scalable and versatile approach for developing lightweight structural components with enhanced performance, suitable for demanding aerospace, automotive, and marine applications.

## Graphical abstract

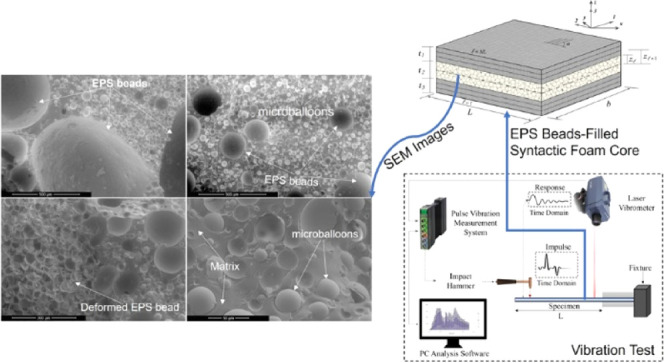

## 1 Introduction

The demand for energy is rapidly and exponentially growing day by day and presenting numerous opportunities in novel, energy-saving and eco-friendly technologies. Such technologies enable significant energy and cost savings through lightweight materials and structures.^1^ At the same time, innovative technologies pave the way for the redesign and modernization of systems with controlled weight and size, while maintaining quality, minimizing costs, preserving durability and structural integrity.^2^ In order to meet these requirements, lightweight materials such as composite foams have proven to be highly advantageous due to their superior strength to weight ratio, ease of processing, and adaptability to various structural applications, making them ideal candidates for modern engineering designs where performance, efficiency, and material economy are critical.^3^ Composite foams are materials with a porous, cell-like structure similar to those found in natural materials like bone, wood, and cork. Structurally, these materials are made by dispersing a large number of voids or cells throughout a continuous matrix which contribute to their functional properties and resulting in low density and unique mechanical behavior.^4^ Among the lightweight composite foams, syntactic foam has a special place due to their excellent features such as mechanical, thermal, electromagnetic, acoustic, vibration and insulation properties.^5^ Hollow spherical particles filling into a binder material (matrix) constructs a new composite called syntactic foams. Polymer, metal, ceramic, bio, and hybrid binders are commonly used for matrix to fabricate syntactic foams.^6^ Hollow particles are made of glass, polymers, ceramic, carbon, metal, and phenolic plastics. The most commonly used particles are glass microspheres due to their ease of production and relatively high strength. Compared to glass microspheres, phenolic plastic ones are commercially available, offer reduced weight, and potentially better adhesion to polymeric matrix, but they lack the strength and hardness of glass.^7^ Despite their widespread use, glass microspheres offer only limited capability in achieving significant weight reduction. However, polymeric fillers such as expanded polystyrene (EPS) and epoxy beads are capable of significantly lowering the density while also enhancing the toughness of syntactic foam.^8–11^

The conventional structure of EPS consists of approximately 98% air and 2% polystyrene material.^12^ It is a lightweight, low-density thermoplastic with small, spherical, closed-cell structures that make it nontoxic, impermeable to fluids, and easy to manufacture in various sizes.^13^ EPS is rigid, stiff, and recyclable, providing excellent energy absorption, thermal and sound insulation, and moisture resistance due to its cellular structure.^14^ These properties enable its broad use in packaging, building insulation, disposable products, protective helmets, and lightweight construction materials.^15,16^ EPS has thus attracted significant research interest, particularly regarding its mechanical behavior. Studies indicate that compressive and tensile strength, as well as elastic modulus, increase with both higher EPS density and strain rate.^17–20^ Density plays a critical role in energy absorption: low-density EPS deforms in a distributed manner, while high-density EPS absorbs more energy through localized cell collapse, albeit with higher force concentration.^21,22^ Under compression, EPS exhibits a distinct stress–strain plateau, where energy is absorbed through cell bending, buckling, or fracture.^14,16,23^ The material also exhibits notable strain-rate sensitivity. At higher loading rates, trapped air within the cells compresses, increasing viscous forces and enhancing stiffness.^24,25^ This results in increased elastic modulus, yield stress, and plateau stress under dynamic conditions.^21,26,27^ EPS effectively dissipates kinetic energy during impact, minimizing force transmission.^28^ This energy absorbing or cushioning behavior has been widely studied both theoretically and experimentally in flexible foams under impact conditions.^29–32^

Technological advancements have enabled composite structures to evolve from secondary roles to primary load-bearing applications. As a result of this transition, these structures are now required to withstand greater loads, necessitating the development of thicker composite configurations. To meet this need, sandwich plates have been developed as a distinct category of thick composite structures. Thanks to their advantages such as high stiffness and strength, excellent energy absorption capability, and superior strength-to-weight ratio, sandwich plates have found increasing applications in aerospace, wind turbine, marine, and civil engineering industries. Accordingly, the development of accurate theoretical formulations is crucial for reliably analyzing sandwich structures.^33^ The analyses of bending, buckling, and free vibrations of sandwich beams using equivalent single-layer theories, layerwise theories, zigzag theories, and exact elasticity approaches have been extensively explored to better understand the mechanical behavior and improve structural performance.^34^ Equivalent single layer theories, including the classical, first, second and third order formulations, polynomial and nonpolynomial displacement methods offer various levels of approximation to capture the through-thickness deformation and stress distributions in sandwich beams and plates.^35^ Equivalent single layer (ESL) theories characterize through-thickness behavior with a single assumed displacement field, which allows their use in analyzing multilayer systems including laminated and sandwich composites.^36^ Higher-order shear deformation theories (HSDTs) can be extended to any desired level of complexity; however, the marginal gain in accuracy often does not justify the increased computational effort and complexity. As a result, third-order theories are generally considered an optimal compromise between accuracy and simplicity. These theories allow the transverse normal deformation to follow second- or third-order curves, eliminating the need for shear correction factors and providing more accurate results.^33^ Additionally, higher-order theories offer improved kinematic representation and more accurate interlaminar stress distribution. The use of cubic terms in the displacement field along the thickness direction allows capturing the second-order variation of transverse shear deformation and stresses across the layers, thereby eliminating the need for shear correction as in the first-order shear deformation theory (FSDT).^37^

In this study, a core structure was developed by combining the advantageous properties of glass microballoons and EPS beads, and a novel sandwich material was designed by integrating this core with a glass fiber composite. To assess their mechanical behavior, the sandwich structures underwent free vibration and flexural testing. Furthermore, a numerical model incorporating third-order shear deformation plate theory was developed and solved via the finite element method to simulate their vibrational characteristics. The agreement between the model and experimental data indicates that the proposed model provides reliable predictive performance. The theoretical framework described above provides the foundation for understanding the mechanical response of the developed sandwich composites. This theoretical approach was essential for accurately modeling the experimental specimens and interpreting their vibrational behavior. Thus, the theoretical and experimental analyses are closely integrated to achieve a comprehensive understanding of the structural performance of the proposed sandwich materials.

## 2 Materials and Methods

### 2.1 Material

The sandwich structure, in this study, mainly consists of glass fiber skins and EPS-filled syntactic foam core. Glass fiber composite skins (GFCS) are manufactured in the laboratory by using vacuum-assisted resin transfer molding (VARTM) technique. Twill-woven glass fibers having 300 g/m^2^ weight were procured from DostKimya Industrial Raw Materials Co. Ltd. (Türkiye). Epoxy resin Duratek DTE 1200 and hardener DTS 1151 are chosen appropriate epoxy system for VARTM and procured from Duratek Protective materials Co. Ltd. (Türkiye). Materials stated above are used for GFCS.

Hollow glass microballoons (HGM), expanded polystyrene (EPS) beads and epoxy resin systems are the constituent materials for core material production. HUNTSMAN Co. branded epoxy resin Araldite GY793 CH and its hardener triethylenetetramine (TETA) are used as binder system and procured from Veser Chemical Materials Inc. (Türkiye). HGMs are supplied by 3 M Ltd. under the trade name of Scotchlite S22, have true particle density 220 kg/m^3^. Three types of EPS beads with different densities are used as fillers. The densities of the EPS beads are 10 kg/m^3^, 18 kg/m^3^ and 30 kg/m^3^. Their diameters range between 3 and 5 mm, 2–3 mm and 1–2 mm, respectively. All component materials selected for this study have been used without any pretreatment.

### 2.2 Methods

#### 2.2.1 Manufacturing of Face Skins

Glass fiber reinforced composite (GFRC) plates are fabricated with VARTM to use as face skins of sandwich structure. Each of face skins have just one layer of glass fiber. The fabrication process of the skins is carried out based on the previous study.^38^ Since only one layer of glass fiber is used, a mixture of 65 g of epoxy and hardener is prepared for the composite layer. Face skins are cut into pieces of the desired sizes from a large GFRC plate with an overall size of 550 × 550 mm^2^. Dimensions of GFRC slabs were 293 mm in length, 25 mm in width and the thickness of 0.35 mm.

#### 2.2.2 Manufacturing of Core Materials

The core of the sandwich composite structure is produced as EPS beads-containing syntactic foam. The matrix material, which has a reduced density due to the inclusion of hollow glass microballoons (HGM), is aimed to be further lightened with additional EPS beads. Typical fabrication procedure of syntactic foams is also performed to manufacture EPS-filled syntactic foam. Three distinct types of hybrid composite cores, along with a neat resin specimen, were fabricated. The volume fractions of the constituents used in the fabrication of the core material were maintained constant, and the corresponding values are presented in Table 1. In the specimen nomenclature, the prefix “c” refers to core material samples, while “s” indicates sandwich composite specimens. Both are followed by the density value of the EPS beads used.

**Table 1:** Material Composition of the EPS-Filled Syntactic Foams and Neat Epoxy Resin

type of cores	EPS density (kg/m^3^)	EPS (vol %)	MB (vol %)	resin system (vol %)
neat epoxy	-	-	-	100
c10	10	50	20	30
c18	18	50	20	30
c30	30	50	20	30

In the initial stage of production, a predetermined quantity of epoxy resin was placed in a glass beaker and heated at 50 °C for 30 min to reduce its viscosity. This step ensured proper wetting of the hollow glass microspheres (HGMs), prevented agglomeration in the mixture, and facilitated the production of a more homogeneous composite. Subsequently, HGMs were gradually incorporated into the resin, and the mixture was manually stirred for 15 min at a slow, controlled pace. Careful stirring was essential to avoid fracturing the microspheres, as their structural integrity is critical to their function. The primary role of HGMs is to reduce the composite’s density by introducing hollow cavities within the matrix. If HGMs fracture during mixing, they fail to perform this function; instead, their hollow cores become filled with epoxy resin, leading to increased composite density rather than a decrease. Furthermore, such breakage compromises the intended porous structure of the material. Once a uniform viscosity was achieved, the mixture was degassed by allowing it to rest for 15 min. Following this, expandable polystyrene (EPS) beads were introduced and gently hand-mixed for an additional 15 min. Finally, the epoxy hardener was incorporated, and mixing continued for 10 min to ensure homogeneous distribution. The prepared mixture was then transferred into an aluminum mold. To further eliminate entrapped air, the mold was subjected to mechanical vibration, minimizing undesired voids. The mold was sealed with a lid and cured at room temperature for 48 h.

#### 2.2.3 Manufacturing of Sandwich Structures

This study examined structural composite sandwich specimens consisting of glass fiber-reinforced polymer (GFRP) face sheets bonded to a syntactic foam core filled with expanded polystyrene (EPS) beads shown in Figure 1. The adhesive system employed Huntsman Araldite AW 106 resin and HV 953U hardener, mixed at a 100:80 weight ratio according to the manufacturer’s specifications. During the bonding process, a uniform compressive load of ∼3 kgf was applied at room temperature to ensure proper adhesion. Upon completion of curing, excess adhesive was carefully removed to maintain dimensional accuracy. The final test specimens measured 293 mm × 25 mm × 15.7 mm (length × width × thickness).

**Figure 1:**
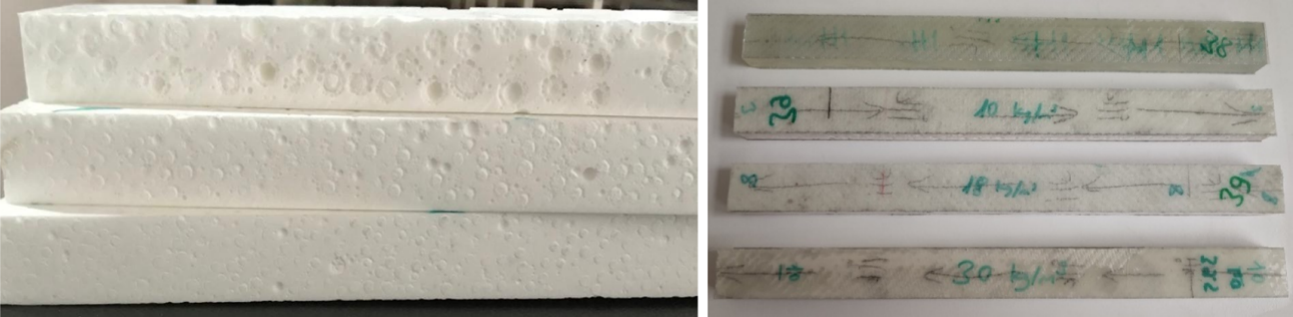
Fabricated EPS bead-filled syntactic foam core and their sandwich form.

#### 2.2.4 Experimental Testing

##### 2.2.4.1 Compression Test

Compression tests were conducted exclusively on the EPS bead-filled syntactic foam composite core materials. The tests were performed using a Shimadzu AGS-X universal testing machine (Shimadzu Corporation) equipped with a 100 kN load cell, in accordance with ASTM C365. The specimens were prepared with dimensions of 25 mm × 25 mm × 15 mm (length × width × thickness). The crosshead speed of the testing machine was set to 1.0 mm/min during the tests.

##### 2.2.4.2 Free Vibration Test

To identify the vibrational properties of the sandwich composite specimens, impact-excited vibration tests were performed using a PULSE analysis system (Brüel & Kjær Sound & Vibration Measurement A/S, Denmark). The fundamental natural frequencies and their corresponding damping ratios of the sandwich structures were systematically measured with respect to various parameters. All specimens were tested using a cantilever-free boundary condition, with uniform dimensions of 212 mm in length and 25 mm in width. The experimental setup, illustrated in Figure 2, consisted of a Brüel & Kjær 3560 fast Fourier transform (FFT) analyzer, a B&K 2302–5 modal impact hammer for excitation, and an Ometron VH300+ laser vibrometer (UK) for noncontact vibration response measurements. Data acquisition was managed via a computer system equipped with the PULSE software platform.^39^ During the tests, the sandwich specimens were excited at specific points using the modal hammer, and the resulting structural responses were recorded by the laser vibrometer. The PULSE software automatically processed the time-domain signals, producing frequency spectra from which the natural frequencies and damping ratios were directly extracted.

**Figure 2:**
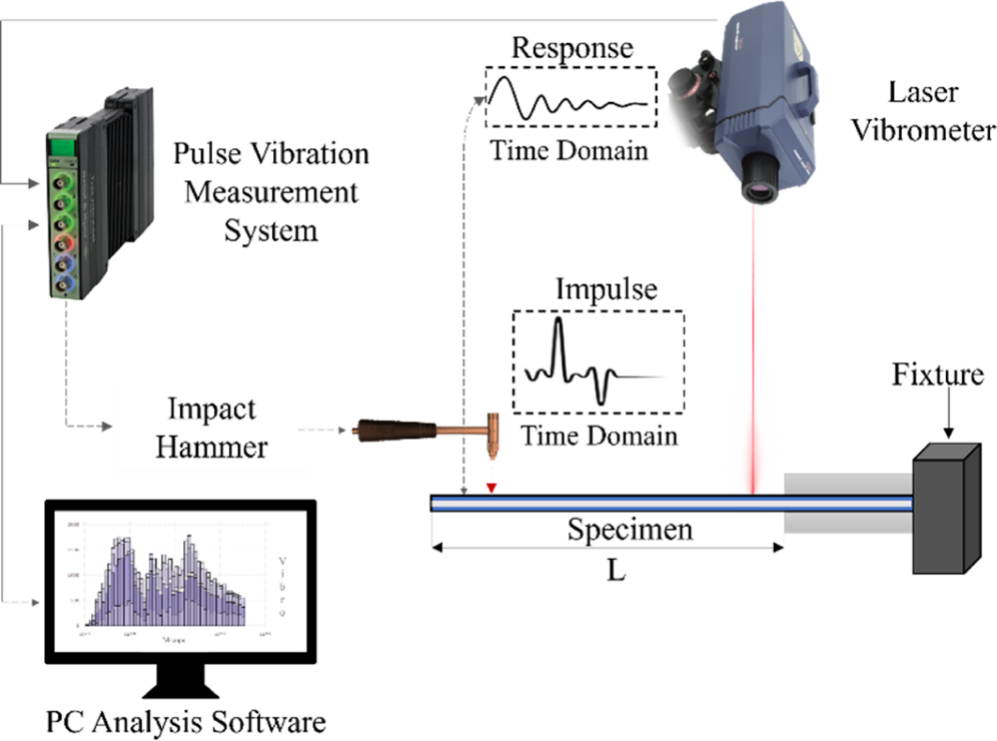
Schematic representation of experimental vibration test setup.

##### 2.2.4.3 Three-Point Bending Test

Three-point bending tests were conducted to evaluate the flexural properties of both core materials and sandwich composites. The experiments were performed using a 100 kN INSTRON-5982 universal testing machine, operating at a constant crosshead speed of 2 mm/min in accordance with ASTM C393 standards. The specimens were designed with a span length of 250 mm, corresponding to a length-to-thickness aspect ratio of 16:1. The core materials had a uniform thickness of 15 mm, while the sandwich composites measured 15.7 mm in thickness. All specimens maintained consistent dimensions of 293 mm in total length and 25 mm in width, ensuring compatibility with the three-point bending test configuration.

#### 2.2.5 Mathematical Formulation

This section presents the finite element methodology developed to numerically analyze the free vibration characteristics of sandwich structures. The mathematical formulation was derived based on third-order shear deformation theory (TSDT) assumptions,^37^ with governing equations implemented in the computational model. Figure 3 illustrates the schematic configuration of the sandwich structure used in the numerical simulations.

$$u\left(x,y,z\right)=u_{o}\left(x,y\right)+z{⌀}_{x}\left(x,y\right)-\frac{4}{3h^{2}}z^{3}\left({⌀}_{x}\left(x,y\right)+\frac{\partial w_{o}}{\partial x}\right)$$

1 $$v\left(x,y,z\right)=v_{o}\left(x,y\right)+z{⌀}_{y}\left(x,y\right)-\frac{4}{3h^{2}}z^{3}\left({⌀}_{y}\left(x,y\right)+\frac{\partial w_{o}}{\partial y}\right)$$

$$w\left(x,y,z\right)=w_{o}\left(x,y,z\right)$$

where *u*, *v* and *w* are displacement components of any point in the laminate element in the *x*, *y*, and *z* directions, respectively. Here *u*_o_, *v*_o_ denote the in-plane displacements, while *w*_o_ refers to the transverse displacement of a point in the midplane. The rotations ⌀_*x*_ and ⌀_*y*_ describe the angular displacements around the *x* and *y* axes, respectively, of lines initially normal to the midplane. The in-plain strain vector $\varepsilon ={\left[\varepsilon_{x}\varepsilon_{y}\gamma_{xy}\right]}^{T}$ can be expressed as

2 $$\varepsilon =\varepsilon_{0}+z\kappa_{1}+z^{3}\kappa_{2}$$

where

3 $$\varepsilon_{0}=\left[\begin{array}{l} \frac{\partial u_{o}}{\partial x}\\ \frac{\partial v_{o}}{\partial y}\\ \frac{\partial u_{o}}{\partial y}+\frac{\partial v_{o}}{\partial x} \end{array}\right],\kappa_{1}=\left[\begin{array}{l} \frac{\partial {⌀}_{x}}{\partial x}\\ \frac{\partial {⌀}_{y}}{\partial y}\\ \frac{\partial {⌀}_{x}}{\partial y}+\frac{\partial {⌀}_{y}}{\partial x} \end{array}\right],\;\kappa_{2}=-\frac{4}{3h^{2}}\left[\begin{array}{l} \frac{\partial {⌀}_{x}}{\partial x}+\frac{\partial \theta_{x}}{\partial x}\\ \frac{\partial {⌀}_{y}}{\partial y}+\frac{\partial \theta_{y}}{\partial y}\\ \frac{\partial {⌀}_{x}}{\partial y}+\frac{\partial {⌀}_{y}}{\partial x}+\frac{\partial \theta_{x}}{\partial y}+\frac{\partial \theta_{y}}{\partial x} \end{array}\right]$$

where θ_*x*_ = ∂*w*_o_/∂*x* and θ_*y*_ = ∂*w*_o_/∂*y* refer to the derivative of *w*_0_ with respect to *x* and *y*. ε contain ε_*x*_, ε_*y*_ and γ_*xy*_ as the in-plain strains (bending strains). The other bending strain form is the transverse shear strain γ consists of γ_*xz*_ and γ_*yz*_. The transverse shear strain vector $\gamma ={\left[\gamma_{xz}\gamma_{yz}\right]}^{T}$ expressed with

4 $$\gamma =\varepsilon_{s}+z^{2}\kappa_{s}$$

where

5 $$\varepsilon_{s}=\left[\begin{array}{l} {⌀}_{x}+\frac{\partial w_{o}}{\partial x}\\ {⌀}_{y}+\frac{\partial w_{o}}{\partial y} \end{array}\right],\kappa_{s}=-\frac{4}{h^{2}}\left[\begin{array}{l} {⌀}_{x}+\theta_{x}\\ {⌀}_{y}+\theta_{y} \end{array}\right]$$

**Figure 3:**
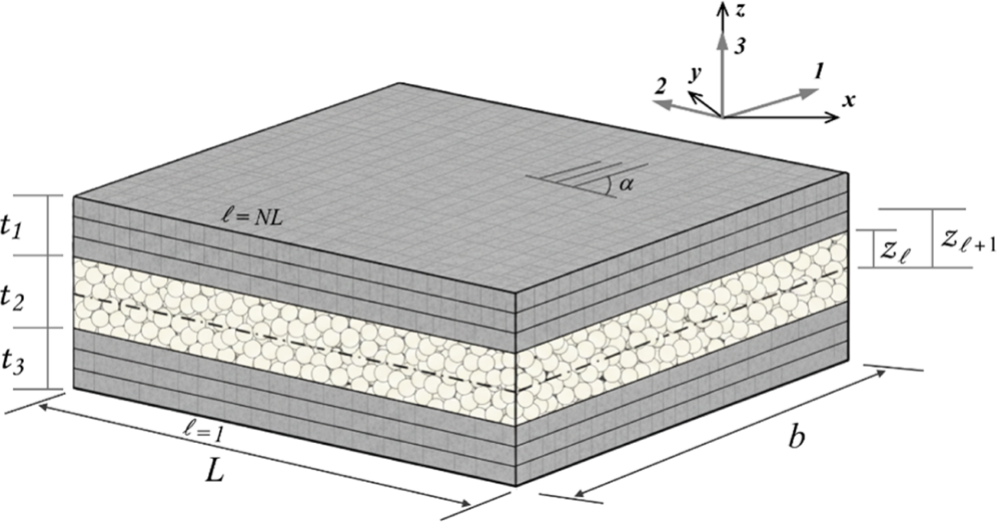
Representative layered composite structure with laminate coordinate system.

As demonstrated in eq 4, the shear stresses exhibit a quadratic variation through the laminate thickness. Consequently, unlike in the first-order shear deformation theory (FSDT), no shear correction factor is required. In the local coordinate system, the constitutive relation for an orthotropic lamina under plane stress conditions can be derived from Hooke’s law and is expressed as follows: γ, the transverse shear strains change quadratically along the laminate thickness. As a result, the shear stresses will also vary quadratically. In this case shear correction factor is not required. For a lamina, the stress–strain relations in the principal material direction 1, 2, and 3 are as follows

6 $${\left\{\begin{array}{l} \sigma_{1}\\ \begin{array}{l} \sigma_{2}\\ \tau_{12}\\ \begin{array}{l} \tau_{13}\\ \tau_{23} \end{array} \end{array} \end{array}\right\}}_{l}={\left[\begin{array}{lllll} C_{11} & C_{12} & 0 & 0 & 0\\ C_{12} & C_{22} & 0 & 0 & 0\\ 0 & 0 & C_{33} & 0 & 0\\ 0 & 0 & 0 & C_{44} & 0\\ 0 & 0 & 0 & 0 & C_{55} \end{array}\right]}_{l}{\left\{\begin{array}{l} \begin{array}{l} \varepsilon_{1}\\ \varepsilon_{2}\\ \gamma_{12} \end{array}\\ \gamma_{13}\\ \gamma_{23} \end{array}\right\}}_{l}$$

where the *C*_*ij*_ ‘s are the plane stress and expressed in terms of material properties. The components of [*C*_*ij*_] are written as

$$C_{11}=\frac{E_{1}}{1-\upsilon_{12}\upsilon_{21}},C_{12}=\frac{{\upsilon_{12}E}_{2}}{1-\upsilon_{12}\upsilon_{21}},C_{22}=\frac{E_{2}}{1-\upsilon_{12}\upsilon_{21}}$$

7 $$C_{33}=G_{12},C_{44}=G_{13},C_{55}=G_{23},\upsilon_{21}E_{1}=\upsilon_{12}E_{2}$$

where E_1_, E_2_ are the Young modulus, G_12_, G_13_, G_23_ are the shear modulus and, υ_12_ and υ_21_ are the Poisson ratios. For a lamina in 1–2 coordinate system needs to be expressed in *x*–*y* coordinate system for the laminated reference system. Accordingly, the stress-strain relation is as follows

8 $${\left\{\begin{array}{l} \sigma_{x}\\ \begin{array}{l} \sigma_{y}\\ \tau_{xy}\\ \begin{array}{l} \tau_{xz}\\ \tau_{yz} \end{array} \end{array} \end{array}\right\}}_{l}={\left[\begin{array}{lllll} \Theta_{11} & \Theta_{12} & \Theta_{13} & 0 & 0\\ \Theta_{12} & \Theta_{22} & \Theta_{23} & 0 & 0\\ \Theta_{13} & \Theta_{23} & \Theta_{33} & 0 & 0\\ 0 & 0 & 0 & \Theta_{44} & \Theta_{45}\\ 0 & 0 & 0 & \Theta_{45} & \Theta_{55} \end{array}\right]}_{l}{\left\{\begin{array}{l} \begin{array}{l} \varepsilon_{x}\\ \varepsilon_{y}\\ \gamma_{xy} \end{array}\\ \gamma_{xz}\\ \gamma_{yz} \end{array}\right\}}_{l}$$

$$\Theta_{11}=C_{11}c^{4}+2\left(C_{12}+2C_{33}\right)c^{2}s^{2}+C_{22}s^{4}$$

$$\Theta_{12}=C_{12}\left(c^{4}+s^{4}\right)+\left(C_{11}+C_{22}-4C_{33}\right)c^{2}s^{2}$$

$$\Theta_{22}=C_{11}s^{4}+2\left(C_{12}+2C_{33}\right)c^{2}s^{2}+C_{22}c^{4}$$

$$\Theta_{13}=\left(C_{11}-C_{12}-2C_{33}\right)sc^{3}+\left(C_{12}-C_{22}+2C_{33}\right)cs^{3}$$

9 $$\Theta_{23}=\left(C_{11}-C_{12}-2C_{33}\right)cs^{3}+\left(C_{12}-C_{22}+2C_{33}\right)sc^{3}$$

$$\Theta_{33}=C_{33}\left(c^{4}+s^{4}\right)+\left(C_{11}+C_{22}-2C_{12}-2C_{33}\right)c^{2}s^{2}$$

$$\Theta_{44}=C_{44}c^{2}+C_{55}s^{2}$$

$$\Theta_{45}=\left(C_{55}-C_{44}\right)cs$$

$$\Theta_{55}=C_{44}s^{2}+C_{55}c^{2}$$

where c = cosα, s = sinα, α is the angle from the *x* axis to the axis 1. The terms Θ_*ij*_ represent the transformed material constants of the lamina. After the deformation, the total strain energy stored in a structural element consists of the sum of the energies of the bending and shear deformation, expressed as

10 $$U=\frac{1}{2}\int_{A}\sum_{l=1}^{NL}\int_{z_{l-1}}^{z_{l}}{\left\{\gamma \right\}}^{T}{\left[\Theta \right]}_{l}\left\{\gamma \right\}dzdA+\frac{1}{2}\int_{A}\sum_{l=1}^{NL}\int_{z_{l-1}}^{z_{l}}{\left\{\varepsilon \right\}}^{T}{\left[\Theta \right]}_{l}\left\{\varepsilon \right\}dzdA$$

Recalling eqs 2 and 4, and by substituting these equations into the eq 10 and integrating with respect to *z* yields

11 $$\begin{array}{l} U=\frac{1}{2}\int_{A}{\left\{\varepsilon_{0}\right\}}^{T}\left[A\right]\left\{\varepsilon_{0}\right\}+{\left\{\varepsilon_{0}\right\}}^{T}\left[B\right]\left\{\kappa_{1}\right\}+{\left\{\varepsilon_{0}\right\}}^{T}\left[E\right]\left\{\kappa_{2}\right\}\\ +{\left\{\kappa_{1}\right\}}^{T}\left[B\right]\left\{\varepsilon_{0}\right\}+{\left\{\kappa_{1}\right\}}^{T}\left[C\right]\left\{\kappa_{1}\right\}+{\left\{\kappa_{1}\right\}}^{T}\left[F\right]\left\{\kappa_{2}\right\}\\ +{\left\{\kappa_{2}\right\}}^{T}\left[E\right]\left\{\varepsilon_{0}\right\}+{\left\{\kappa_{2}\right\}}^{T}\left[F\right]\left\{\kappa_{1}\right\}+{\left\{\kappa_{2}\right\}}^{T}\left[H\right]\left\{\kappa_{2}\right\}dA\\ +\frac{1}{2}\int_{A}{\left\{\varepsilon_{s}\right\}}^{T}\left[A_{s}\right]\left\{\varepsilon_{s}\right\}+{\left\{\varepsilon_{s}\right\}}^{T}\left[C_{s}\right]\left\{\kappa_{s}\right\}+{\left\{\kappa_{s}\right\}}^{T}\left[C_{s}\right]\left\{\varepsilon_{s}\right\}+{\left\{\kappa_{s}\right\}}^{T}\left[F_{s}\right]\left\{\kappa_{s}\right\}dA \end{array}$$

where [A] , [B] , [C] , [E] , [F] , [H] , $\left[A_{s}\right]$ , $\left[C_{s}\right]$ and $\left[F_{s}\right]$ are given as follows

12 $$\begin{array}{l} \left[A\right]=\sum_{l=1}^{NL}{\left[\Theta_{b}\right]}_{l}\left(z_{l}-z_{l-1}\right),\left[B\right]=\frac{1}{2}\sum_{l=1}^{NL}{\left[\Theta_{b}\right]}_{l}\left({z_{l}}^{2}-{z_{l-1}}^{2}\right)\\ \left[C\right]=\frac{1}{3}\sum_{l=1}^{NL}{\left[\Theta_{b}\right]}_{l}\left({z_{l}}^{3}-{z_{l-1}}^{3}\right),\left[E\right]=\frac{1}{4}\sum_{l=1}^{NL}{\left[\Theta_{b}\right]}_{l}\left({z_{l}}^{4}-{z_{l-1}}^{4}\right)\\ \left[F\right]=\frac{1}{5}\sum_{l=1}^{NL}{\left[\Theta_{b}\right]}_{l}\left({z_{l}}^{5}-{z_{l-1}}^{5}\right),\left[H\right]=\frac{1}{7}\sum_{l=1}^{NL}{\left[\Theta_{b}\right]}_{l}\left({z_{l}}^{7}-{z_{l-1}}^{7}\right)\\ \left[A_{s}\right]=\sum_{l=1}^{NL}{\left[\Theta_{s}\right]}_{l}\left(z_{l}-z_{l-1}\right),\left[C_{s}\right]=\frac{1}{3}\sum_{l=1}^{NL}{\left[\Theta_{s}\right]}_{l}\left({z_{l}}^{3}-{z_{l-1}}^{3}\right)\\ \left[F_{s}\right]=\frac{1}{5}\sum_{l=1}^{NL}{\left[\Theta_{s}\right]}_{l}\left({z_{l}}^{5}-{z_{l-1}}^{5}\right) \end{array}$$

The eq 11 can be expressed in the closed form as

13 $$U=\frac{1}{2}{\left\{D\right\}}^{T}\left\{\int_{A}\left[{\left[B\right]}^{T}\left[\mathrm{D̅}\right]\left[B\right]+{\left[B_{s}\right]}^{T}\left[D_{s}\right]\left[B_{s}\right]\right]dA\right\}\left\{D\right\}$$

where **D̅** and **D**_s_ are material constant matrices equal to

14 $$\mathrm{D̅}=\left[\begin{array}{lll} A & B & E\\ B & C & F\\ E & F & H \end{array}\right],D_{s}=\left[\begin{array}{ll} A_{s} & C_{s}\\ C_{s} & F_{s} \end{array}\right]$$

The area integral in eq 13 equals to the global stiffness matrix (K), which can be expressed as

15 $$K=\int_{A}\left\{{\left[B\right]}^{T}\left[\mathrm{D̅}\right]\left[B\right]+{\left[B_{s}\right]}^{T}\left[D_{s}\right]\left[B_{s}\right]\right\}dA$$

The strain energy can be written as a function of the stiffness matrix as

16 $$U=\frac{1}{2}{\left\{D\right\}}^{T}\left[K\right]\left\{D\right\}$$

Recalling the definitions of ε_0_, κ_1_, κ_2_, ε_s_ and κ_s_ which are presented eqs 3 and 4, these can be expressed as

17 $$\left\{\begin{array}{l} \varepsilon_{0}\\ \kappa_{1}\\ \kappa_{2} \end{array}\right\}=\left[L_{1}\right]\left\{\varepsilon_{xy}\right\}$$

where

18 $$\left\{\varepsilon_{xy}\right\}=\left\{u_{0,x}u_{0,y}v_{0,x}v_{0,y}{⌽}_{x,x}{⌽}_{x,y}{⌽}_{y,x}{⌽}_{y,y}\theta_{x,x}\theta_{x,y}\theta_{y,x}\theta_{y,y}\right\}$$

19 $$\left\{\begin{array}{l} \varepsilon_{s}\\ \kappa_{s} \end{array}\right\}=\left[\Gamma_{1}\right]\left\{\gamma_{xy}\right\}$$

where

20 $$\left\{\gamma_{xy}\right\}=\left\{w_{0,x}w_{0,y}{⌽}_{x}{⌽}_{y}\theta_{x}\theta_{y}\right\}$$

The terms {ε_*xy*_} and {γ_*xy*_}, which are defined depending on the derivatives related to *x* and *y*, need to be expressed as derivatives of *s* and *t*. This transformation can be done by the help of Jacobian matrix [J], which establish a relation between the derivatives of two coordinate systems as follows

21 $$T={\left[J\right]}^{-1}=\frac{1}{J}\left[\begin{array}{ll} \frac{\partial y}{\partial t} & -\frac{\partial y}{\partial s}\\ -\frac{\partial x}{\partial t} & \frac{\partial x}{\partial s} \end{array}\right]$$

After the definition of Jacobian matrix, {ε_*xy*_} and {γ_*xy*_} can be written as

22 $$\left\{\varepsilon_{xy}\right\}=\left[\begin{array}{lllll} T & 0 & 0 & 0 & .\\ 0 & T & 0 & 0 & .\\ 0 & 0 & T & 0 & .\\ 0 & 0 & 0 & T & .\\ . & . & . & . & . \end{array}\right]\left\{\begin{array}{l} u_{0,s}\\ u_{0,t}\\ v_{0,s}\\ .\\ . \end{array}\right\}=\left[L_{2}\right]\left\{\varepsilon_{st}\right\}$$

23 $$\left\{\gamma_{xy}\right\}=\left[\begin{array}{ll} T & 0\\ 0 & I \end{array}\right]\left\{\gamma_{st}\right\}=\left[\Gamma_{2}\right]\left\{\gamma_{st}\right\}$$

where, [*I*] is the identity matrix.

24 $$\left\{\varepsilon_{st}\right\}=\left[L_{3}\right]\left\{D\right\},\left\{\gamma_{st}\right\}=\left[\Gamma_{3}\right]\left\{D\right\}$$

­[L_1_], [L_2_], [L_3_], [Γ_1_], [Γ_2_], and [Γ_3_] are defined and presented in the appendix. For the final step, we need to express {ε} and {γ} in terms of the nodal degrees of freedom by

25 $$\left[B\right]=\left[L_{1}\right]\left[L_{2}\right]\left[L_{3}\right],\left[B_{s}\right]=\left[\Gamma_{1}\right]\left[\Gamma_{2}\right]\left[\Gamma_{3}\right]$$

For the dynamic analysis the mass matrix is required. The mass matrix of the laminated composite plate can be obtained from the expression of the kinetic energy of the plate.

26 $$T=\frac{1}{2}\int_{A}\sum_{k=1}^{n}\int_{z_{k-1}}^{z_{k}}\rho_{k}\left\{({\mathrm{u̇}}^{2}+{\mathrm{v̇}}^{2}+{ẇ}^{2})\right\}dzdA$$

The derivatives of the displacements in eq 1 with respect to the time are substituted in eq 26 gives

27 $$T=\frac{1}{2}\int_{A}\sum_{k=1}^{n}\int_{z_{k-1}}^{z_{k}}\rho_{k}\left\{\begin{array}{l} \left({\mathrm{u̇}}_{0}^{2}+{\mathrm{v̇}}_{0}^{2}+{ẇ}_{0}^{2}\right)+\left(z^{2}+2cz^{4}+c^{2}z^{6}\right)\left({⌽̇}_{x}^{2}+{⌽̇}_{y}^{2}\right)\\ +c^{2}z^{6}\left({\mathrm{\theta ̇}}_{x}^{2}+{\mathrm{\theta ̇}}_{y}^{2}\right)+2\left(z+cz^{3}\right)\left({⌽̇}_{x}{\mathrm{u̇}}_{0}+{⌽̇}_{y}{\mathrm{v̇}}_{0}\right)\\ +2cz^{3}\left({\mathrm{\theta ̇}}_{x}{\mathrm{u̇}}_{0}+{\mathrm{\theta ̇}}_{y}{\mathrm{v̇}}_{0}\right)+2\left(cz^{4}+c^{2}z^{6}\right)\left({⌽̇}_{x}{\mathrm{\theta ̇}}_{x}+{⌽̇}_{y}{\mathrm{\theta ̇}}_{y}\right) \end{array}\right\}dzdA$$

If displacements are assumed to be time-dependent harmonic functions, each of the displacements can be supposed to be premultiplied by e^iωt^. Straight after, the time derivatives can be expressed as the displacements themselves multiplied by iω, as indicated in the appendix. To facilitate the development of the mass matrix, substituting the expressions defined in the appendix into eq 26 and integrating in the *z*-direction yields the following equation.

28 $$T=-\frac{1}{2}\omega^{2}\int_{A}{\left\{d\right\}}^{T}\sum_{k=1}^{n}\int_{z_{k-1}}^{z_{k}}\rho_{k}\left\{[\begin{array}{l} H_{1}+e_{1}\left[H_{2}\right]+e_{2}\left[H_{3}\right]\\ +e_{3}\left[H_{4}\right]+e_{4}\left[H_{5}\right]+e_{5}\left[H_{6}\right] \end{array}]\right\}\left\{d\right\}dzdA$$

­[H_i_] and e_i_ are also defined in the appendix.

In the present study, a nine-node isoparametric element with seven nodal unknowns per node is used.^37^ Hence, each element has a total of 63 degrees of freedom. The continuum displacement vector {d} = {u v w**⌽**_*x*_ **⌽**_*y*_θ_*x*_θ_*y*_} at any point on the midsurface is defined by {d} = [N]­{D} where *N* is the interpolating function associated with node i. The final form for T follows after writing {d} in terms of {D} and shape functions: {d} = [N]­{D} and its transpose {d}^T^ = {D}^T^[N] we have

29 $$T=-\frac{1}{2}\omega^{2}\int_{A}{\left\{D\right\}}^{T}\left\{[\begin{array}{l} {\left[N\right]}^{T}\left[{\mathrm{H̅}}_{1}\right]\left[N\right]+{\left[N\right]}^{T}\left[{\mathrm{H̅}}_{2}\right]\left[N\right]+{\left[N\right]}^{T}\left[{\mathrm{H̅}}_{3}\right]\left[N\right]\\ +{\left[N\right]}^{T}\left[{\mathrm{H̅}}_{4}\right]\left[N\right]+{\left[N\right]}^{T}\left[{\mathrm{H̅}}_{5}\right]\left[N\right]+{\left[N\right]}^{T}\left[{\mathrm{H̅}}_{6}\right]\left[N\right] \end{array}]\right\}\left\{D\right\}dA$$

where

30 $$\left[{\mathrm{H̅}}_{i}\right]=R_{i}\left[H_{i}\right],i=1,2,...,6$$

and

$$R_{1}=\sum_{k=1}^{n}\rho_{k}\left(t_{k}-t_{k-1}\right)$$

$$R_{2}=\sum_{k=1}^{n}\rho_{k}\left(\frac{t_{k}^{3}-t_{k-1}^{3}}{3}+\frac{2c}{5}\left(t_{k}^{5}-t_{k-1}^{5}\right)+\frac{c^{2}}{7}\left(t_{k}^{7}-t_{k-1}^{7}\right)\right)$$

31 $$R_{3}=\sum_{k=1}^{n}\rho_{k}\frac{c^{2}}{7}\left(t_{k}^{7}-t_{k-1}^{7}\right)$$

$$R_{4}=\sum_{k=1}^{n}\rho_{k}\left(\frac{t_{k}^{2}-t_{k-1}^{2}}{2}+\frac{c}{4}\left(t_{k}^{4}-t_{k-1}^{4}\right)\right)$$

$$R_{5}=\sum_{k=1}^{n}\rho_{k}\frac{c}{4}\left(t_{k}^{4}-t_{k-1}^{4}\right)$$

$$R_{6}=\sum_{k=1}^{n}\rho_{k}\left(\frac{c}{5}\left(t_{k}^{5}-t_{k-1}^{5}\right)+\frac{c^{2}}{7}\left(t_{k}^{7}-t_{k-1}^{7}\right)\right)$$

The eq 29 can be rewritten in the closed form as

32 $$T=-\frac{1}{2}\omega^{2}{\left\{D\right\}}^{T}\left[M\right]\left\{D\right\}$$

where [*M*] is the mass matrix. After determining the U and T , Lagrangian functional L=U−T can be calculated for the system. If the Hamilton principle $\delta \int_{t_{1}}^{t_{2}}\left(U-T\right)dt$ is applied to the Lagrangian functional, finite element equation of the system is derived. From now on, the eigenvalue equation for determining the natural frequencies of the free vibration of the system can be easily obtained as follows.

33 $$\left(\left[K\right]-\omega^{2}\left[M\right]\right)\left\{D\right\}=0$$

where ω is the natural frequency and {D} is the displacement vector.

## 3 Results and Discussions

### 3.1 Analysis of Microscopy

Scanning electron microscopy (SEM) analysis was carried out to investigate the microstructural characteristics of the fabricated composites (Figure 4). The SEM images reveal that the constituent materials are uniformly and homogeneously distributed within the matrix. A strong interfacial bonding between the hollow glass microspheres (HGMs), expanded polystyrene (EPS) bead fillers, and the matrix resin is clearly observed. From the fracture surface, both deformed (after fracture) and undeformed EPS beads were present within the composite structure. The internal cellular morphology of the deformed EPS beads is distinctly visible, indicating their structural response under processing or loading conditions. Notably, the matrix resin did not penetrate the interiors of the EPS beads, preserving their lightweight functionality. At higher magnification, it was confirmed that the HGMs remained intact during the manufacturing process. The preservation of their spherical morphology is critical, as any breakage would lead to infiltration by the matrix, thereby increasing mass and reducing the overall efficiency of the composite. Maintaining the structural integrity of the HGMs ensures they fulfill their intended role in enhancing mechanical performance while maintaining low density.

**Figure 4:**
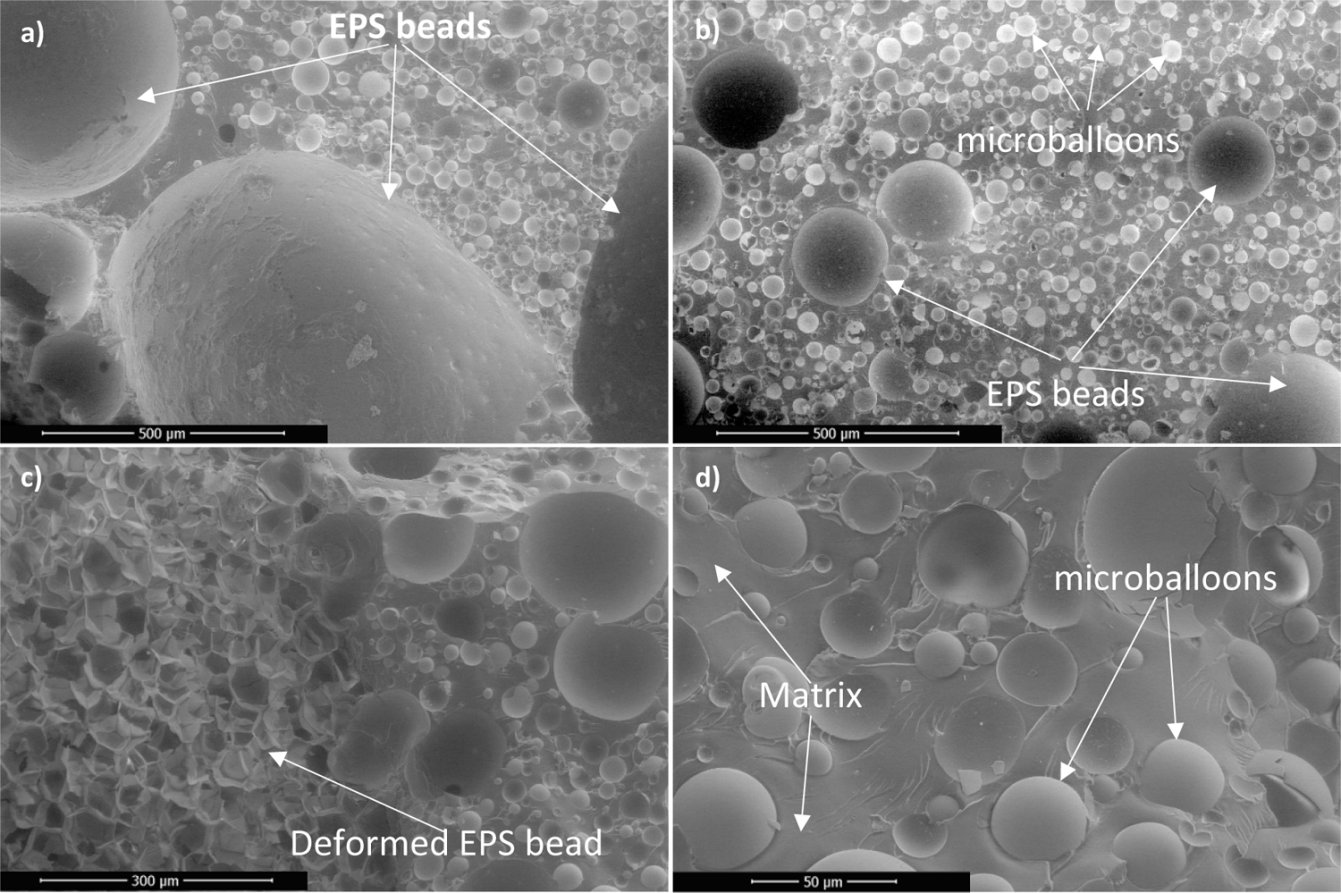
SEM micrographs of the EPS beads filled-syntactic foam. (a) c10 sample (250 X mag) (b) c18 sample (250 X mag) (c) c30 sample (400 X mag) (d) syntactic foam structure (1500 X mag).

### 3.2 Analysis of Density

Experimental measurements were conducted to determine the density of the single-ply glass fiber reinforced composite material. The results indicated that the density of the glass fiber reinforced composites employed as the face sheets in the sandwich structure is 1.700 g/cm^3^.

The densities of the fabricated slabs—comprising neat resin, EPS-filled syntactic foams with varying EPS bead densities, and their corresponding sandwich structures—were measured and are summarized in Table 2. As previously stated, the quantity of hollow glass microspheres (HGMs) was held constant in the mixture, while the bulk density of EPS beads served as the sole variable parameter in the composition. Consequently, increasing the density of EPS beads in the composite formulation results in a corresponding elevation in the overall composite density. This inverse relationship between particle size and material density demonstrates that employing larger EPS beads produces lower-density syntactic foams. Although syntactic foams are primarily composed of hollow glass microspheres (HGMs), significant challenges remain in achieving ultralow-density materials using HGMs due to their inherent material limitations.

**Table 2:** Densities of the Fabricated Slabs

density of structures (kg/m^3^)	neat resin (kg/m^3^)	EPS bulk density (kg/m^3^)		
		10	18	30
core materials (cNE, c10, c18, c30)	1166.77	470.73	482.68	483.12
sandwich materials (sNE, s10, s18, s30)	1190.03	543.82	554.21	554.69

Gupta et al.^40,41^ reported a density of 493 kg/m^3^ and 549 kg/m^3^ for a syntactic foam made of HGMs/epoxy resin containing 65 vol.% and 60 vol.% microballoons (ρ_MB_ = 220 kg/m^3^), respectively. Similarly, Gupta et al.^42^ stated a density of 554.7 kg/m^3^ for a syntactic foam made of HGMs/vinyl ester resin containing of 60 vol.% microballoons (ρ_MB_ = 220 kg/m^3^). The results indicate that the relatively high density of the microballoons themselves is the limiting factor in achieving a further reduction in the overall density of the structure, despite their high volume fraction of 60–65%. In this study, under the 20 vol.% MB, 50 vol.% EPS beads and 30 vol % resin conditions, densities of slabs were measured 470.73 kg/m^3^ for (ρ_EPS_ = 10 kg/m^3^), 482.68 kg/m^3^ for (ρ_EPS_ = 18 kg/m^3^) and 483.12 kg/m^3^ for (ρ_EPS_ = 30 kg/m^3^). When comparing the obtained density results with those reported in the literature, it is evident that the use of ultralow density polymeric EPS beads can serve as effective and promising fillers for producing lower-density materials. Moreover, further optimization may be achieved by adjusting the production method, filler particle size, and filler volume fraction, as demonstrated in.^43^

### 3.3 Compressive Properties of Foam Cores

Compression tests were conducted on syntactic foam composites reinforced with expanded polystyrene (EPS) beads, which were fabricated as core materials. For each composite type, four samples were tested, and the average values are provided in the tables and figures. The stress-strain curves obtained from these tests (Figure 5a) reveal distinct mechanical behavior across composites produced with varying EPS bead densities and neat epoxy (Figure 5b). As illustrated in Figure 5, the stress–strain response of EPS bead-filled syntactic foam exhibits two characteristic regions: the first is a linear-elastic phase with a steep slope, corresponding to elastic deformation of the foam structure; the second is a plateau region, indicative of progressive bead crushing and material densification. The compressive strength of the composites showed a direct correlation with EPS bead density (Figure 6a). Specifically, the c30 composite, fabricated with EPS beads of 30 kg/m^3^ density, demonstrated the highest compressive strength (6.63 MPa). This was followed by the c18 (4.81 MPa) and c10 (4.37 MPa) composites, produced with 18 kg/m^3^ and 10 kg/m^3^ EPS beads, respectively. The observed trend—c10 < c18 < c30—clearly indicates that increasing the EPS bead density enhances compressive strength. This phenomenon can be attributed to the higher structural integrity imparted by denser EPS beads, which reduce void content and improve load transfer efficiency within the epoxy matrix. Furthermore, the mechanical behavior of these syntactic foams was compared with that of the neat epoxy matrix (cNE), which exhibited significantly higher compressive strength (79.16 MPa) due to the absence of porosity (Figure 6b). The syntactic foams, however, displayed a more gradual failure mechanism characterized by bead collapse and energy absorption, making them suitable for applications requiring controlled deformation under load. The relationship between compressive strength and composite density was further analyzed, reinforcing the conclusion that smaller EPS beads (higher density) contribute to greater stiffness and strength compared to larger beads. This relation can be expressed with the visual evidence in Figure 7 strongly supports the deformation pattern of the produced composites. While the c10 specimen shown in Figure 7a exhibited a high tendency for disintegration under compressive loading due to the inability of the beads to maintain structural integrity, the c30 specimen shown in Figure 7b was able to sustain higher loads by preserving the cohesion of its smaller bead network, even in the presence of crack propagation. This aligns with established theories on particulate composites, where filler size and distribution critically influence mechanical performance. Smaller beads increase interfacial adhesion and reduce stress concentrations, thereby delaying crack initiation. These findings are consistent with prior research,^43^ confirming that syntactic foams with higher-density EPS beads exhibit superior compressive properties. The study underscores the importance of microstructural optimization in syntactic foam design, where bead density serves as a key parameter for tailoring mechanical performance.

**Figure 5:**
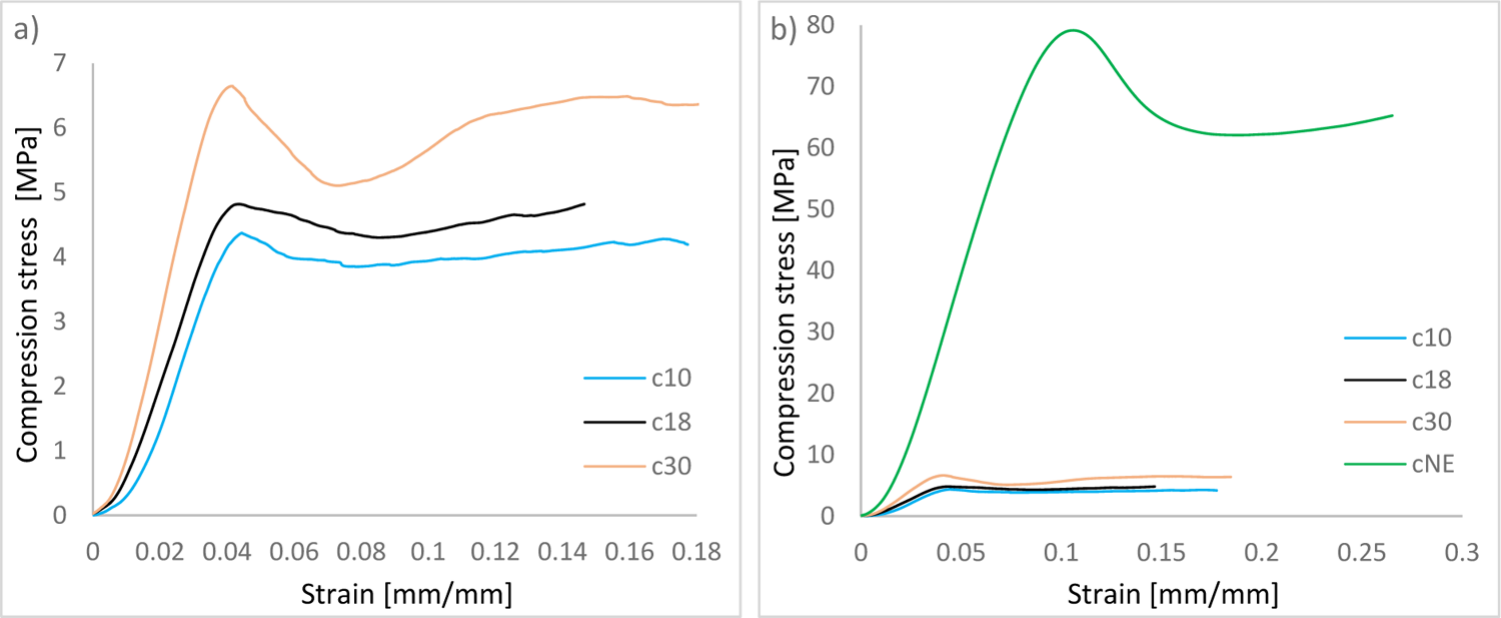
Compressive stress-strain curves of fabricated core materials (a) without (b) with cNE.

**Figure 6:**
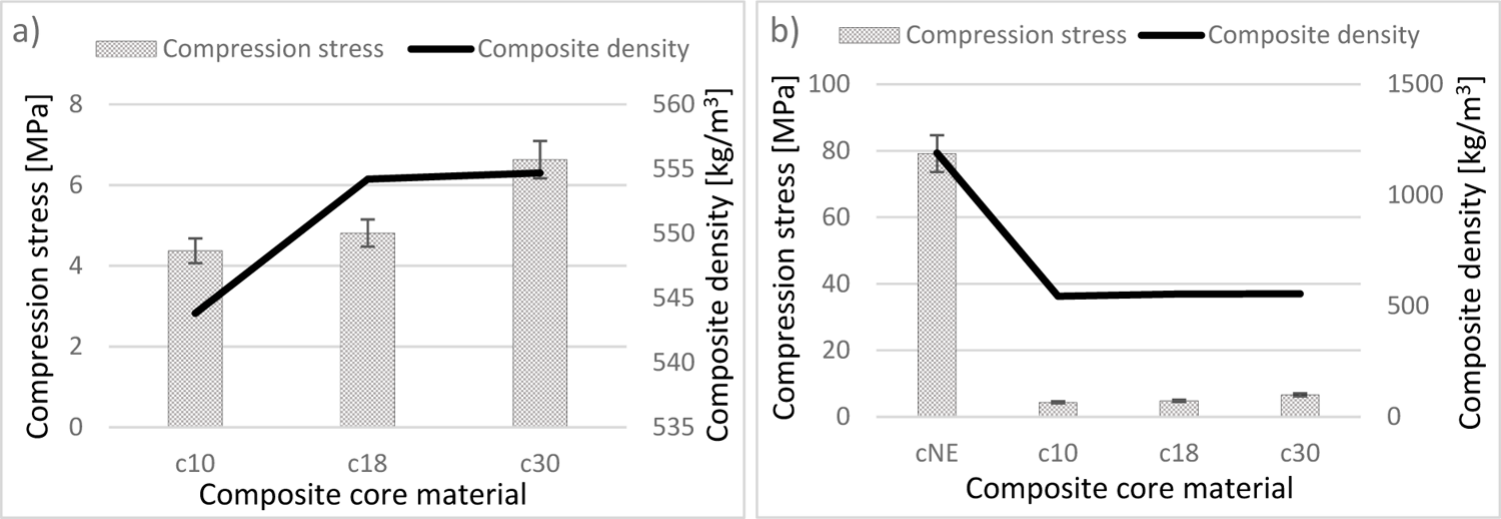
Compressive stress values versus density of the core composites (a) without (b) with cNE.

**Figure 7:**
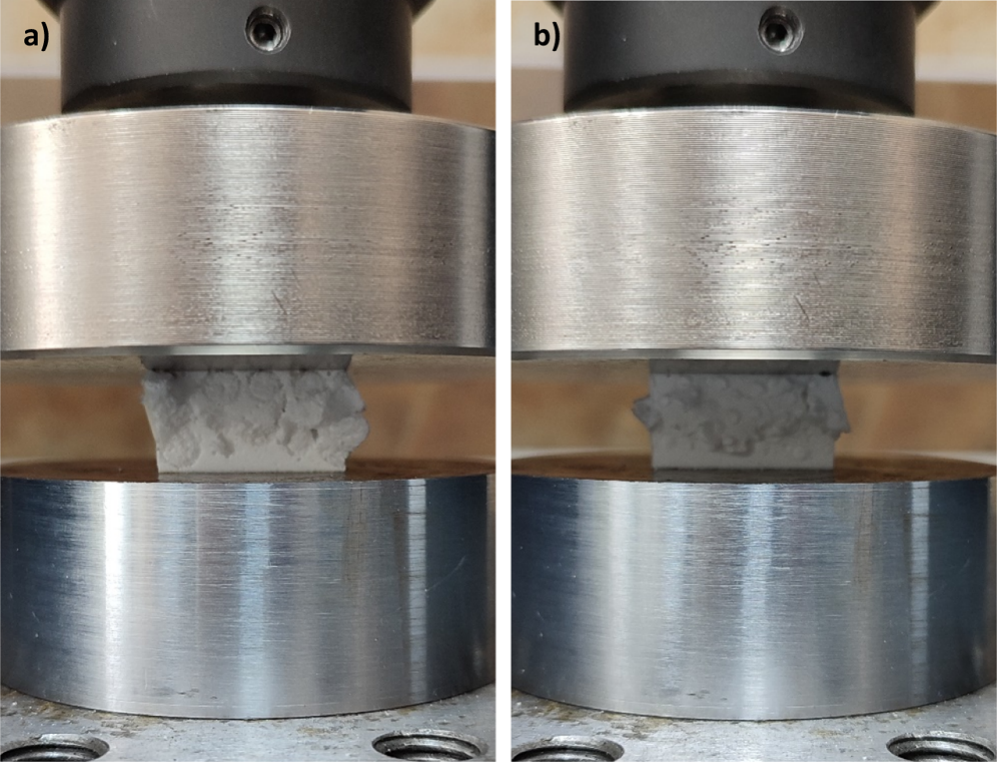
EPS bead-filled syntactic foam failure during compression test, (a) c10 and (b) c30.

Additionally, the plateau region observed in the syntactic foams is indicative of progressive bead collapse and structural densification, which contributes to gradual energy absorption under compressive loading. This deformation mode contrasts with the brittle fracture of the neat epoxy and highlights the advantage of syntactic foams in applications where controlled deformation and damage tolerance are essential.

### 3.4 Vibration Characteristics of Sandwich Composites

This study presents an investigation into the vibration behavior of sandwich composites incorporating syntactic foam cores reinforced with expanded polystyrene (EPS) beads, the mechanical properties of which are summarized in Table 3, utilizing both experimental and numerical methods. Free vibration tests conducted under fixed-free boundary conditions revealed systematic variations in the natural frequencies and damping ratios of the specimens. As depicted in Table 4, the neat epoxy sample (sNE) exhibited the lowest fundamental frequency (103.83 Hz), whereas the EPS-filled counterparts demonstrated progressively higher values: 129.66 Hz (s10), 133.00 Hz (s18), and 139.00 Hz (s30). The steady increase in natural frequency with rising EPS bead density indicates that the enhancement in structural stiffness—achieved through reduced void content and more efficient load transfer in the composite core—becomes more dominant than the accompanying increase in mass. In contrast, damping ratios showed a declining trend with increasing EPS content. The highest damping was observed in the neat epoxy specimen (1.690%), while EPS-reinforced samples exhibited lower values: 0.784% (s10), 0.715% (s18), and 0.670% (s30). This decrease is primarily attributed to diminished viscoelastic energy dissipation in the stiffer EPS-filled cores and reduced interfacial friction due to more densely packed filler particles. The reduction in bead size appears to facilitate more efficient vibration transmission while concurrently restricting conventional damping mechanisms. These results highlight a fundamental trade-off between stiffness and damping in EPS-based sandwich composites. The findings offer valuable guidance for optimizing the design of lightweight structural elements where control over vibrational response and energy dissipation is critical.

**Table 3:** Mechanical Properties of the Composite Materials

material	Ex (GPa)	Ey (GPa)	Ez (GPa)	Gxy (GPa)	Gyz (GPa)	Gxz (GPa)	ν_*xy*_	ν_*yz*_	ν_*xz*_	ρ (kg/m^3^)
glass fiber	5.233	5.233	3.400	2.013	2.013	1.307	0.3	0.3	0.3	1700
EPS 10	0.970	0.970	0.970	0.360	0.360	0.360	0.35	0.35	0.35	470.73
EPS 18	1.062	1.062	1.062	0.393	0.393	0.393	0.35	0.35	0.35	482.68
EPS 30	1.088	1.088	1.088	0.403	0.403	0.403	0.35	0.35	0.35	483.12
neat epoxy	1.653	1.653	1.653	0.621	0.621	0.621	0.33	0.33	0.33	1166.77

**Table 4:** Free Vibration Results for Fixed-Free Boundary Condition

sandwich structure	first natural frequency (Hz)		damping ratio (%)
	experimental	numerical	experimental
sNE	103.83	109.72	1.690
s10	129.66	132.10	0.784
s18	133.00	135.60	0.715
s30	139.00	136.92	0.670

Figure 8a illustrates the relationship between composite density and the natural frequencies of syntactic foam-core sandwich composites fabricated with EPS beads. As the density of EPS beads increases, both the composite density and natural frequencies show a corresponding rise. When correlated with bead size, it becomes evident that decreasing EPS bead size—while increasing their bulk density—results in higher composite densities and natural frequencies. Despite the increase in mass due to higher EPS bead content, the observed rise in natural frequencies suggests a more significant enhancement in structural stiffness. According to the relation ω = √(k/m), this implies that the stiffness increment outweighs the mass increase, leading to elevated natural frequencies. Figure 8c further compares the EPS-reinforced syntactic foam composites (s10, s18, s30) with the neat epoxy sandwich composite (sNE) in terms of natural frequency and overall density. The sNE specimen exhibits the lowest natural frequency, despite having the highest composite density. This behavior is attributed to its relatively heavier structure, where the influence of mass surpasses that of stiffness in determining dynamic response. Specifically, the density of the sNE sample is 1190.03 kg/m^3^, while the s10, s18, and s30 samples exhibit significantly reduced values of 543.83 kg/m^3^ (54.30% decrease), 554.21 kg/m^3^ (53.42% decrease), and 554.69 kg/m^3^ (53.39% decrease), respectively. The substantial mass reduction in these foam-core structures enhances their dynamic performance, as evidenced by their higher natural frequencies.

**Figure 8:**
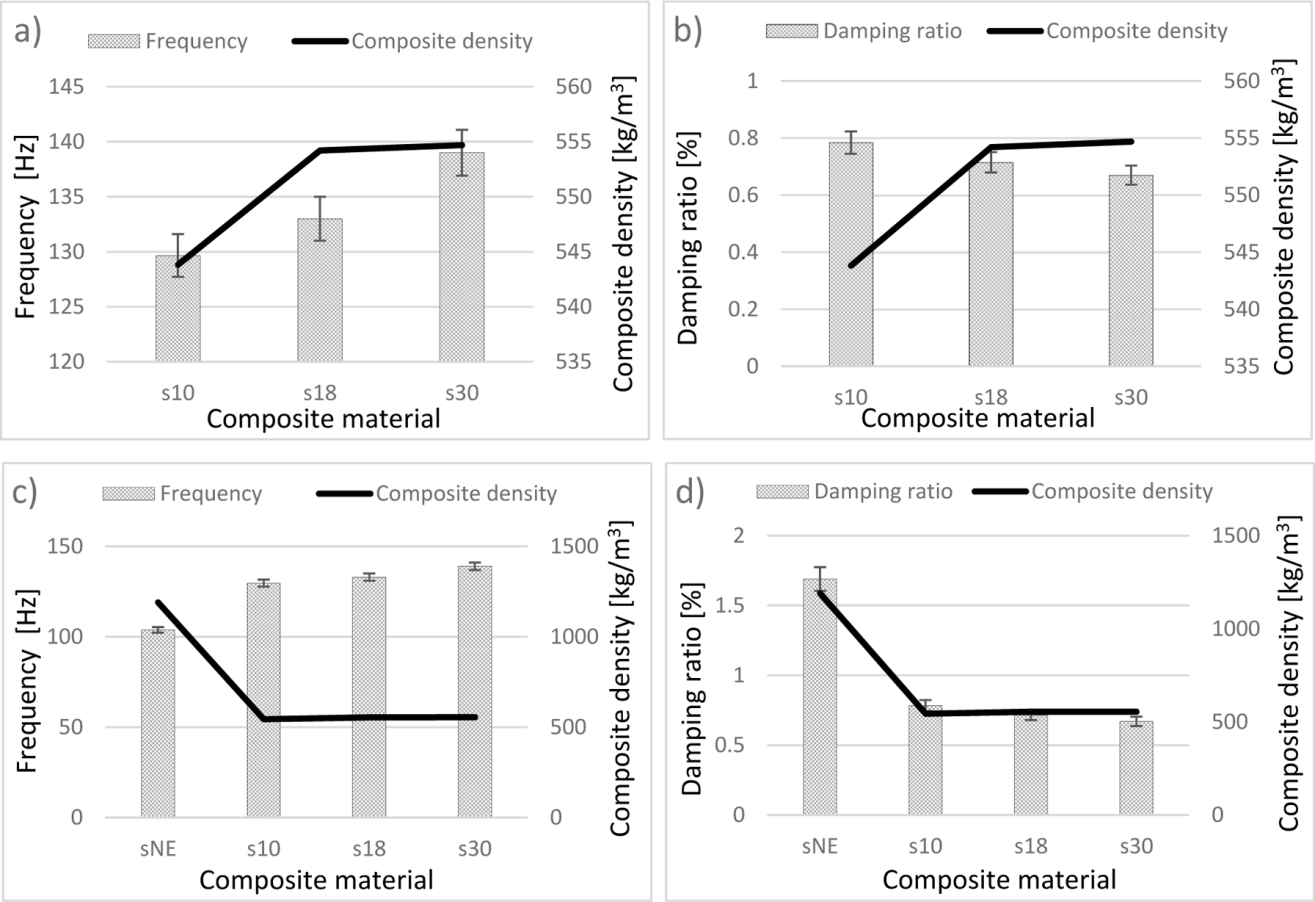
Variations of composite densities versus the natural frequencies (a) without sNE (c) with sNE and the damping ratios of sandwich structures (b) without sNE (d) with sNE.

The relationship between EPS bead density and damping behavior in foam-core sandwich composites is presented in Figure 8b, based on experimental data. A clear trend emerges: as the density of the EPS beads increases, the damping ratio decreases. Specifically, the s10 samples, which incorporate the lowest-density and largest-sized EPS beads, achieved the highest damping ratio at 0.784%. This value dropped to 0.715% in the s18 samples and further declined to 0.67% in the s30 samples. These results suggest that the damping performance of foam-core composites is significantly influenced by the size and density of the embedded EPS beads. Larger beads—typically associated with lower bulk density—enhance the material’s ability to dissipate mechanical energy. In contrast, smaller beads (i.e., those found in higher-density EPS formulations) appear to restrict internal movement, leading to reduced energy dissipation and thus lower damping values. Interestingly, although the s18 and s30 composites exhibit similar overall densities, the damping ratio of the s30 samples is noticeably lower. This indicates that bead size, rather than composite density alone, plays a dominant role in determining damping characteristics. Further insight is provided in Figure 8d, which compares the damping performance of foam-core composites with that of a neat epoxy sandwich structure (sNE). Among all samples, sNE demonstrates the highest damping ratio, surpassing even the best-performing foam-core configuration (s10). This superior performance is attributed to the absence of internal voids in the neat epoxy core, which facilitates greater internal friction and, therefore, more effective energy dissipation. Overall, the findings highlight the importance of carefully selecting EPS bead characteristics when designing sandwich composites for dynamic applications. While lower-density, larger-sized EPS beads enhance damping capacity, higher-density beads may be more suitable in applications where stiffness and higher natural frequencies are prioritized.

### 3.5 Flexural Properties of Sandwich Composites

The fabricated core foam composite materials (Figure 9a,b) and the glass fiber sandwich composite structures incorporating these foam cores (Figure 9c) were evaluated using three-point bending tests. The results of these tests, presented in Figure 9a, demonstrate the influence of expanded polystyrene (EPS) bead density on flexural performance. Among the tested specimens, the syntactic foam composite reinforced with EPS beads at a density of 30 kg/m^3^ (designated as c30) exhibited the highest flexural strength, reaching 12.49 MPa. This was followed by the c18 composite (EPS density: 18 kg/m^3^) with a flexural strength of 12.09 MPa, and the c10 composite (EPS density: 10 kg/m^3^) with a strength of 10.72 MPa. These findings indicate a direct correlation between EPS bead density and mechanical performance—higher-density EPS beads contribute to increased flexural strength and stiffness. Furthermore, the study revealed that smaller EPS bead sizes, at equivalent volume fractions, enhance flexural properties. This improvement is attributed to the greater interfacial interaction and stress distribution within the composite matrix when finer beads are employed. Additionally, c10 specimens exhibited greater deformation capacity compared to c18 and c30, suggesting that lower-density EPS beads yield composites with higher ductility. Consequently, for applications requiring enhanced deformability, the use of larger, lower-density EPS beads may be preferable. For comparative analysis, Figure 9b illustrates the flexural behavior of EPS bead-filled syntactic foam composites against neat epoxy (cNE), the base polymer matrix used in composite fabrication. The neat epoxy specimen demonstrated a significantly higher flexural strength (66.03 MPa), owing to its continuous, cross-linked thermoset structure, which ensures superior mechanical integrity. In contrast, the introduction of EPS beads and glass microballoons (GMB) disrupts structural homogeneity, leading to localized stress concentrations and reduced mechanical performance. Additionally, despite stringent processing controls, the inadvertent entrapment of air voids during manufacturing further diminishes strength. These factors collectively account for the marked difference in flexural performance between cNE and the EPS bead-reinforced composites (c10, c18, c30), as depicted in Figure 9b.

**Figure 9:**
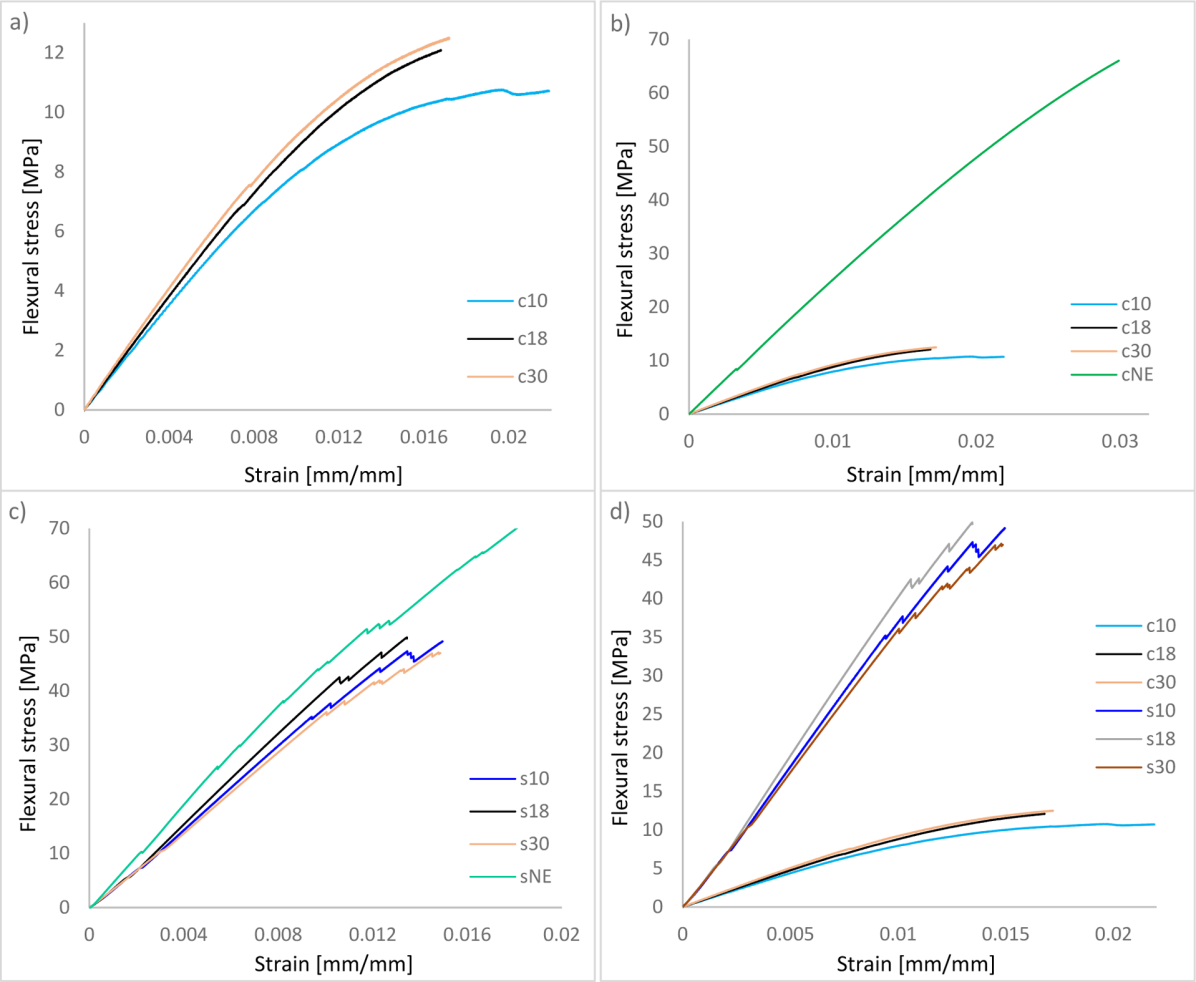
Flexural stress-strain curves of fabricated core materials (a) without cNE (b) with cNE and their sandwich forms (c) all sandwich composites (d) comparison of cores and sadwich forms.

Figure 9c illustrates the bending behavior of hybrid syntactic foam core sandwich composites—fabricated using EPS beads with varying densities—in comparison with the neat epoxy core sandwich composite structure under three-point bending tests. The flexural stress values of the fabricated sandwich composites were measured as 70.45 MPa for the sNE sample (with a neat epoxy core), 49.13 MPa for s10, 49.66 MPa for s18, and 46.98 MPa for s30. Among the foam-core sandwich structures (s10, s18, and s30), no significant differences in flexural performance were observed, as both stress and strain values remained relatively consistent. In contrast, the sNE specimen demonstrated a markedly higher flexural strength compared to all EPS-modified core structures.

Interestingly, the pronounced stress disparity observed between the neat epoxy and EPS-based syntactic foams (as shown in Figure 9b) is substantially diminished in the sandwich composite configurations (Figure 9c). Despite utilizing the same core materials, the stress difference becomes less significant once these cores are integrated into sandwich structures. This highlights the pivotal role of the composite face sheets—specifically, the single-layer glass fiber reinforcement—and the adhesive interface in governing the overall flexural behavior.

A closer analysis reveals that the face sheets contribute more dominantly to the flexural response than the core materials. As evidenced in Figure 9b, the variation in stress and deformation across syntactic foam cores with different EPS bead densities remains minimal. This finding suggests that although the core materials are inherently weak due to their low density and mechanical strength, their incorporation into a sandwich configuration substantially improves structural efficiency. A similar reinforcing effect can also be observed in Figure 9d. Expanded polystyrene (EPS) bead-filled syntactic foam composites exhibit flexural strengths in the range of approximately 10–12.5 MPa when tested in isolation. However, upon integration into sandwich structures with single-layer glass fiber-reinforced composite face sheets, their flexural performance improves significantly—by approximately a factor of 5—reaching values between 46.98 and 49.13 MPa. These findings highlight the considerable potential of low-density EPS-filled syntactic foams as core materials for lightweight sandwich composite applications.

## 4 Conclusion

This study presents the development of a novel sandwich composite material consisting of an EPS bead-filled syntactic foam core bonded to glass fiber-reinforced composite face sheets. The fabricated sandwich structures were experimentally evaluated through free vibration and flexural tests. Additionally, a numerical model was developed to analyze the vibrational behavior of the structure based on third-order shear deformation plate theory (TSDT), with solutions obtained via the finite element method (FEM). Furthermore, the core material alone was subjected to compression testing to assess its mechanical properties independently. The key findings of this study are summarized as follows.

- Density characteristics: experimental results demonstrated a clear positive relationship between EPS bead density and core material density. This correlation can also be interpreted as showing that core density increases with decreasing EPS bead diameter, as smaller beads allow for more efficient packing within the composite matrix.
- Vibration test findings: vibration analysis revealed that natural frequencies showed a significant increase with higher EPS bead densities. This trend indicates that structures incorporating smaller diameter EPS beads exhibit greater vibrational stiffness. The close agreement between experimental natural frequency measurements and numerical predictions validated the accuracy of the implemented third-order shear deformation plate theory (TSDT) finite element model.
- Damping behavior: in contrast to the frequency results, damping ratios exhibited an inverse relationship with bead density. Specifically, composites with larger EPS beads demonstrated superior energy dissipation characteristics, suggesting that bead size plays a critical role in vibration damping performance.
- Flexural properties: flexural testing showed consistent improvements in both strength and stiffness with increasing EPS bead density. These mechanical enhancements were similarly observed when comparing specimens with progressively smaller bead diameters, confirming the dual influence of density and size effects.
- Deformation capacity analysis: comparative evaluation revealed that specimens containing low-density (larger diameter) EPS beads permitted substantially greater deformation before failure. This characteristic makes them particularly suitable for applications where energy absorption or impact resistance is prioritized.
- Structural enhancement through sandwich design: while unreinforced syntactic foam cores displayed limited flexural resistance (10–12.5 MPa), their incorporation into sandwich structures with glass fiber-reinforced face sheets produced a remarkable 5-fold increase in bending stress resistance (46.98–49.13 MPa), demonstrating the effectiveness of this structural configuration.
- Compressive performance: compression testing established a direct proportionality between EPS bead density and compressive strength. This relationship further manifested as improved load-bearing capacity with decreasing bead size, highlighting the importance of microstructural characteristics in determining mechanical performance.

## Appendix

$$e_{1}=z^{2}+2cz^{4}+c^{2}z^{6},e_{2}=c^{2}z^{6},e_{3}=2\left(z+cz^{3}\right),e_{4}=2cz^{3},e_{5}=2\left(cz^{4}+c^{2}z^{6}\right)$$

$$\left\{\begin{array}{l} {\mathrm{u̇}}_{0}\\ {\mathrm{v̇}}_{0}\\ {ẇ}_{0}\\ 0\\ 0\\ 0\\ 0 \end{array}\right\}=i\omega \left[\begin{array}{lllllll} 1 & 0 & 0 & 0 & 0 & 0 & 0\\ 0 & 1 & 0 & 0 & 0 & 0 & 0\\ 0 & 0 & 1 & 0 & 0 & 0 & 0\\ 0 & 0 & 0 & 0 & 0 & 0 & 0\\ 0 & 0 & 0 & 0 & 0 & 0 & 0\\ 0 & 0 & 0 & 0 & 0 & 0 & 0\\ 0 & 0 & 0 & 0 & 0 & 0 & 0 \end{array}\right]\left\{\begin{array}{l} u_{0}\\ v_{0}\\ w_{0}\\ {⌽}_{x}\\ {⌽}_{y}\\ \theta_{x}\\ \theta_{y} \end{array}\right\}=i\omega \left[H_{1}\right]\left\{d\right\}$$

$$\left\{\begin{array}{l} 0\\ 0\\ 0\\ {⌽̇}_{x}\\ {⌽̇}_{y}\\ 0\\ 0 \end{array}\right\}=i\omega \left[\begin{array}{lllllll} 0 & 0 & 0 & 0 & 0 & 0 & 0\\ 0 & 0 & 0 & 0 & 0 & 0 & 0\\ 0 & 0 & 0 & 0 & 0 & 0 & 0\\ 0 & 0 & 0 & 1 & 0 & 0 & 0\\ 0 & 0 & 0 & 0 & 1 & 0 & 0\\ 0 & 0 & 0 & 0 & 0 & 0 & 0\\ 0 & 0 & 0 & 0 & 0 & 0 & 0 \end{array}\right]\left\{\begin{array}{l} u_{0}\\ v_{0}\\ w_{0}\\ {⌽}_{x}\\ {⌽}_{y}\\ \theta_{x}\\ \theta_{y} \end{array}\right\}=i\omega \left[H_{2}\right]\left\{d\right\}$$

$$\left\{\begin{array}{l} 0\\ 0\\ 0\\ 0\\ 0\\ {\mathrm{\theta ̇}}_{x}\\ {\mathrm{\theta ̇}}_{y} \end{array}\right\}=i\omega \left[\begin{array}{lllllll} 0 & 0 & 0 & 0 & 0 & 0 & 0\\ 0 & 0 & 0 & 0 & 0 & 0 & 0\\ 0 & 0 & 0 & 0 & 0 & 0 & 0\\ 0 & 0 & 0 & 0 & 0 & 0 & 0\\ 0 & 0 & 0 & 0 & 0 & 0 & 0\\ 0 & 0 & 0 & 0 & 0 & 1 & 0\\ 0 & 0 & 0 & 0 & 0 & 0 & 1 \end{array}\right]\left\{\begin{array}{l} u_{0}\\ v_{0}\\ w_{0}\\ {⌽}_{x}\\ {⌽}_{y}\\ \theta_{x}\\ \theta_{y} \end{array}\right\}=i\omega \left[H_{3}\right]\left\{d\right\}$$

$$\left\{\begin{array}{l} {⌽̇}_{x}\\ {⌽̇}_{y}\\ 0\\ 0\\ 0\\ 0\\ 0 \end{array}\right\}=i\omega \left[\begin{array}{lllllll} 0 & 0 & 0 & 1 & 0 & 0 & 0\\ 0 & 0 & 0 & 0 & 1 & 0 & 0\\ 0 & 0 & 0 & 0 & 0 & 0 & 0\\ 0 & 0 & 0 & 0 & 0 & 0 & 0\\ 0 & 0 & 0 & 0 & 0 & 0 & 0\\ 0 & 0 & 0 & 0 & 0 & 0 & 0\\ 0 & 0 & 0 & 0 & 0 & 0 & 0 \end{array}\right]\left\{\begin{array}{l} u_{0}\\ v_{0}\\ w_{0}\\ {⌽}_{x}\\ {⌽}_{y}\\ \theta_{x}\\ \theta_{y} \end{array}\right\}=i\omega \left[H_{4}\right]\left\{d\right\}$$

$$\left\{\begin{array}{l} {\mathrm{\theta ̇}}_{x}\\ {\mathrm{\theta ̇}}_{y}\\ 0\\ 0\\ 0\\ 0\\ 0 \end{array}\right\}=i\omega \left[\begin{array}{lllllll} 0 & 0 & 0 & 0 & 0 & 1 & 0\\ 0 & 0 & 0 & 0 & 0 & 0 & 1\\ 0 & 0 & 0 & 0 & 0 & 0 & 0\\ 0 & 0 & 0 & 0 & 0 & 0 & 0\\ 0 & 0 & 0 & 0 & 0 & 0 & 0\\ 0 & 0 & 0 & 0 & 0 & 0 & 0\\ 0 & 0 & 0 & 0 & 0 & 0 & 0 \end{array}\right]\left\{\begin{array}{l} u_{0}\\ v_{0}\\ w_{0}\\ {⌽}_{x}\\ {⌽}_{y}\\ \theta_{x}\\ \theta_{y} \end{array}\right\}=i\omega \left[H_{5}\right]\left\{d\right\}$$

$$\left\{\begin{array}{l} 0\\ 0\\ 0\\ {\mathrm{\theta ̇}}_{x}\\ {\mathrm{\theta ̇}}_{y}\\ 0\\ 0 \end{array}\right\}=i\omega \left[\begin{array}{lllllll} 0 & 0 & 0 & 0 & 0 & 0 & 0\\ 0 & 0 & 0 & 0 & 0 & 0 & 0\\ 0 & 0 & 0 & 0 & 0 & 0 & 0\\ 0 & 0 & 0 & 0 & 0 & 1 & 0\\ 0 & 0 & 0 & 0 & 0 & 0 & 1\\ 0 & 0 & 0 & 0 & 0 & 0 & 0\\ 0 & 0 & 0 & 0 & 0 & 0 & 0 \end{array}\right]\left\{\begin{array}{l} u_{0}\\ v_{0}\\ w_{0}\\ {⌽}_{x}\\ {⌽}_{y}\\ \theta_{x}\\ \theta_{y} \end{array}\right\}=i\omega \left[H_{6}\right]\left\{d\right\}$$

$$L_{1}=\left[\begin{array}{llllllllllll} 1 & 0 & 0 & 0 & 0 & 0 & 0 & 0 & 0 & 0 & 0 & 0\\ 0 & 0 & 0 & 1 & 0 & 0 & 0 & 0 & 0 & 0 & 0 & 0\\ 0 & 1 & 1 & 0 & 0 & 0 & 0 & 0 & 0 & 0 & 0 & 0\\ 0 & 0 & 0 & 0 & 1 & 0 & 0 & 0 & 0 & 0 & 0 & 0\\ 0 & 0 & 0 & 0 & 0 & 0 & 0 & 1 & 0 & 0 & 0 & 0\\ 0 & 0 & 0 & 0 & 0 & 1 & 1 & 0 & 0 & 0 & 0 & 0\\ 0 & 0 & 0 & 0 & 1 & 0 & 0 & 0 & 0 & 0 & 0 & 0\\ 0 & 0 & 0 & 0 & 0 & 0 & 0 & 1 & 0 & 0 & 0 & 1\\ 0 & 0 & 0 & 0 & 0 & 1 & 1 & 0 & 0 & 1 & 1 & 0 \end{array}\right]$$

$$\Gamma_{1}=\left[\begin{array}{llllll} 1 & 0 & 1 & 0 & 0 & 0\\ 0 & 1 & 0 & 1 & 0 & 0\\ 0 & 0 & 3 & 0 & 3 & 0\\ 0 & 0 & 0 & 3 & 0 & 3 \end{array}\right]$$

$$L_{3}=\left[\begin{array}{lllllllllll} N_{1,s} & 0 & 0 & 0 & 0 & 0 & 0 & N_{2,s} & 0 & \cdot \cdot \cdot & 0\\ N_{1,t} & 0 & 0 & 0 & 0 & 0 & 0 & N_{2,t} & 0 & \cdot \cdot \cdot & 0\\ 0 & N_{1,s} & 0 & 0 & 0 & 0 & 0 & 0 & N_{2,s} & \cdot \cdot \cdot & 0\\ 0 & N_{1,t} & 0 & 0 & 0 & 0 & 0 & 0 & N_{2,t} & \cdot \cdot \cdot & 0\\ \vdots & & & & & & & \ddots & & & \vdots \\ \vdots & & & & & & & & & \ddots & \\ 0 & 0 & 0 & 0 & 0 & 0 & 0 & N_{1,t} & \cdot \cdot \cdot & \cdot \cdot \cdot & N_{2,t} \end{array}\right]$$

$$\Gamma_{3}=\left[\begin{array}{lllllllllll} 0 & 0 & N_{1,s} & 0 & 0 & 0 & 0 & 0 & 0 & N_{2,s} & \cdot \cdot \cdot \\ 0 & 0 & N_{1,t} & 0 & 0 & 0 & 0 & 0 & 0 & N_{2,t} & \cdot \cdot \cdot \\ 0 & 0 & 0 & N_{1} & 0 & 0 & 0 & 0 & 0 & 0 & \cdot \cdot \cdot \\ 0 & 0 & 0 & 0 & N_{1} & 0 & 0 & 0 & 0 & 0 & \cdot \cdot \cdot \\ 0 & 0 & 0 & 0 & 0 & N_{1} & 0 & 0 & 0 & 0 & \cdot \cdot \cdot \\ 0 & 0 & 0 & 0 & 0 & 0 & N_{1} & 0 & 0 & 0 & \cdot \cdot \cdot \end{array}\right]$$
